# The TCL1A Oncoprotein Interacts Directly with the NF-κB Inhibitor IκB

**DOI:** 10.1371/journal.pone.0006567

**Published:** 2009-08-10

**Authors:** Virginie Ropars, Gilles Despouy, Marc-Henri Stern, Serge Benichou, Christian Roumestand, Stefan T. Arold

**Affiliations:** 1 CNRS, UMR5048, Centre de Biochimie Structurale, Montpellier, France; 2 INSERM, U554, Montpellier, France; 3 Universités Montpellier 1 & 2, Montpellier, France; 4 Institut Curie, Centre de Recherche, Paris, France; 5 INSERM U830, Paris, France; 6 Institut Cochin, Université Paris Descartes, CNRS, UMR 8104, Inserm U567, Paris, France; 7 Inserm U567, Paris, France; University of Queensland, Australia

## Abstract

The T cell leukaemia/lymphoma 1A (TCL1A) oncoprotein plays key roles in several B and T cell malignancies. Lacking enzymatic activity, TCL1A's transforming action was linked to its capacity to co-activate the protein kinase AKT *via* binding to its pleckstrin homology (PH) domain. However, perturbation of AKT signalling alone was recently shown insufficient to explain TCL1A oncogenesis, suggesting that TCL1A has additional cellular partners. Searching for such additional targets, we found that TCL1A binds specifically and directly to the ankyrin domain of IκB, the inhibitor of the NF-κB transcription factors. Through binding assays and a structural analysis by small angle X-ray scattering, we show that TCL1A and IκB interact in yeast-two-hybrid systems, when transiently overexpressed in 293 cells, and as recombinant proteins *in vitro*. We further establish that the association between TCL1A and IκB is compatible with AKT binding to TCL1A, but incompatible with IκB binding to NF-κB. By interfering with the inhibition of NF-κB by IκB, TCL1A may increase the concentration of free NF-κB molecules sufficiently to trigger expression of anti-apoptotic genes. Thus our data suggest an additional route by which TCL1A might cause cancer.

## Introduction

TCL1-family proteins (TCL1A, TCL1B and MTCP1 in humans) are 13–15 kDa non-enzymatic interaction modules. Their restricted physiological expression and apparent lack of auto-regulatory mechanisms suggest that they are mainly regulated by tightly controlled gene expression levels. Normal time- and tissue-restricted expression of *TCL1* genes is important for efficient fertility, and the development of T and B cell lineages. Deregulation of *TCL1* expression can cause the transformation of mature human T and B lymphocytes, and is associated with a variety of human diseases, including T-cell prolymphocytic leukaemia, B-cell chronic leukaemia (B-CLL), B-cell lymphoma and germ-cell tumours [reviewed in [1], [2]].

TCL1A is a co-activator of AKT, a serine/threonine protein kinase that regulates many cellular processes, including proliferation and survival [3], [4]. TCL1A forms homodimers, and each protomer binds one pleckstrin homology (PH) domain of AKT [5]. Co-activation may thus result from stabilising AKT in an open (active) conformation, from promoting AKT auto-phosphorylation in *trans*, and/or from reinforcing AKT attachment to the membrane, where its effectors are located [1], [2]. Activated AKT can promote cell survival by phosphorylating downstream targets such as BAD [6]. AKT can also activate the NF-κB transcription factor, possibly by phosphorylating the IκB kinase (IKK)[7]. Phosphorylated IKK phosphorylates IκB, leading to dissociation of the IκB:NF-κB complex. Liberated NF-κB subsequently translocates to the nucleus to activate survival-inducing genes [8].

However, the effects of TCL1A on AKT are insufficient to fully explain TCL1A oncogenesis. For example, AKT phosphorylation or inactivation kinetics do not consistently relate to TCL1A expression in both transgenic mice and patient–derived neoplasia, and AKT activation alone does not necessarily cause tumours in B cells [1]. This suggests that additional effects, and effectors, of TCL1A exist. Recently, it was reported that the NF-κB pathway is important for B-CLL in transgenic mouse models (reviewed in [9]), and that TCL1A activates NF-κB through an AKT-independent route [10]. Corroborating an AKT-independent targeting of NF-κB by TCL1A, we here report that TCL1A binds directly to the NF-κB inhibitor IκB.

## Results

**Figure 1:**
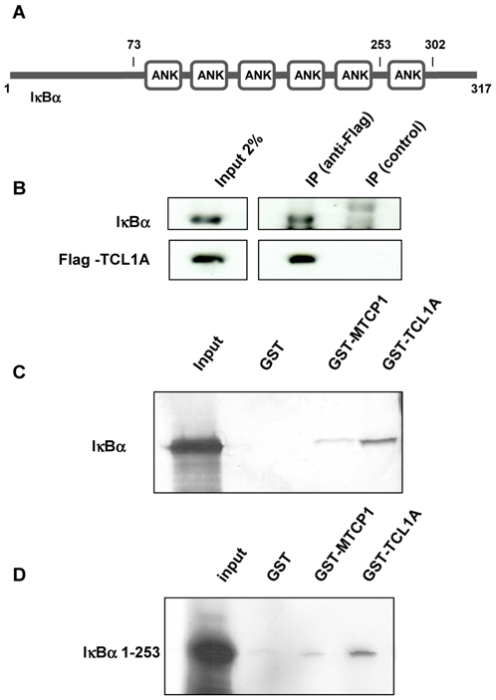
Cell-based binding assays. (a) Schematic representation of IκBα. (b) Co-immunoprecipitation assay. Input 2%: 2% of the whole extract used for the immunoprecipitation. IP control: Immunoprecipitation with irrelevant antibody (anti-HA). (c,d) [^35^S]-labeled IκBα or IκBα_1–253_ produced by *in vitro* transcription/translation in rabbit reticulocytes lysate, were incubated with GST, GST-MTCP1 and GST-TCL1A immobilized on glutathione-sepharose beads. Bound proteins were resolved by 12% SDS-PAGE and detected by autoradiography.

In a search for additional partners of TCL1A, we identified IκBα through yeast two-hybrid screening. The prey consisted of residues 73–317 of IκBα, and included the six ankyrin repeats that bind NF-κB [11], [12] (Figure 1a). After over-expression of IκBα and FLAG-TCL1A in 293 cells, IκBα coimmunoprecipitated with TCL1A, demonstrating that these proteins can interact in a cellular context (Figure 1b). GST-TCL1A interacted significantly more strongly with IκBα than did GST-MTCP1 (Figure 1c). Conversely, two-hybrid, gel shift, and fluorescence anisotropy assays failed to detect an interaction between MTCP1 and IκBα (data not shown). *In vitro*, GST-TCL1A bound the AKTβ PH domain (AKT_PH; residues 1–118), and an all-cysteine-to-serine (7CS) mutant of the IκBα ankyrin domain (IκB_ank, residues 67–302; the C->S substitutions were made to avoid non-specific aggregation through cys-cys di-sulphide bridges), but not the ankyrin domain of the *Drosophila* signalling protein Notch (Figure S1a).

**Figure 2:**
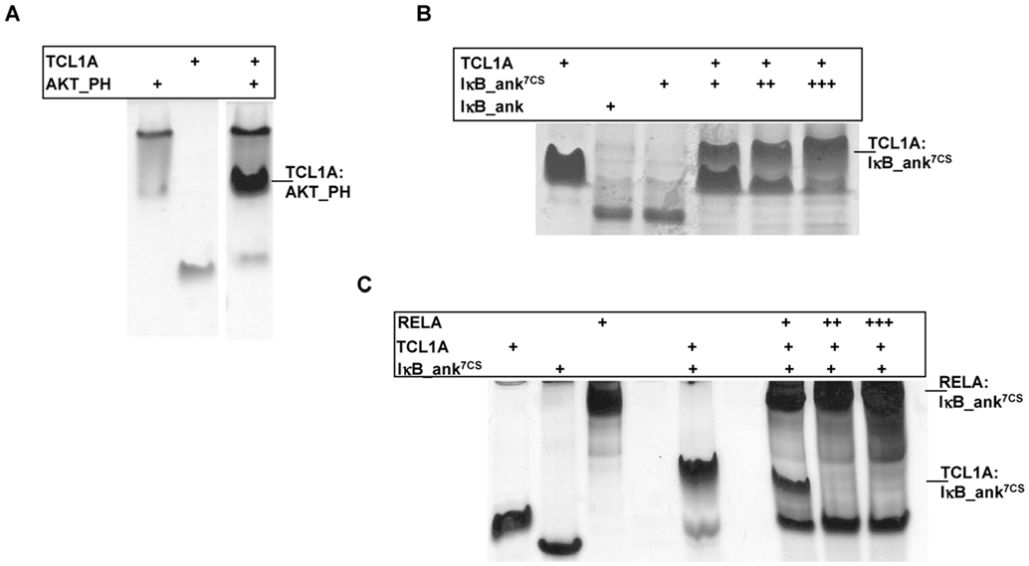
Native gel shift assays. (a–c): Bands corresponding to the complexes formed are indicated on the right of each figure. (b,c): The three lanes on the right correspond to increasing concentrations of IκB_ank^7CS^ (b) or RELA (c).

**Figure 3:**
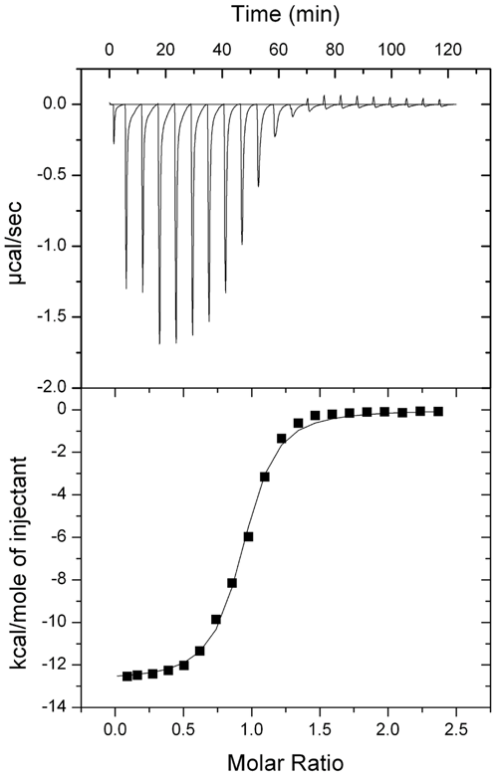
ITC analysis. TCL1A at 470 µM was injected into the measurement cell containing IκB_ank^7CS^ at 45 µM. The titration was carried out at 20°C. The upper panel shows the binding isotherm, the lower panels shows the integrated heats.

Native SDS PAGE gel shift assays showed that AKT_PH, IκB_ank and IκB_ank^7CS^ retarded migration of TCL1A (Figure 2, Figure S1b,c). Using isothermal titration calorimetry (ITC), TCL1A displayed a dissociation constant (K_D_) of 7.5±1.0 µM for AKT_PH, in agreement with previous NMR measurements [5] (Table 1, and Figure S2a). TCL1A bound IκB_ank^7CS^ with a K_D_ of 0.71±0.07 µM (Figure 3). Placing the 1∶1 TCL1A∶IκB_ank^7CS^ complex in the ITC cell slightly increased the affinity of injected AKT_PH (K_D_ = 5.1±0.5 µM), as compared to titrations with only TCL1A in the cell. This showed that binding of IκB_ank and AKT_PH to TCL1A are compatible and slightly cooperative events (Figure S2b). Conversely, when the cell was filled with IκB_ank^7CS^ bound to the NF-κB family member RELA, injection of TCL1A yielded curves with a K_D_≫50 µM, inferring that binding of TCL1A and RELA to IκB_ank are incompatible events (data not shown). Competition between TCL1A and RELA for IκB_ank was confirmed by gel shift assays (Figure 2c).

**Table 1:** Summary of ITC data.

Syringe/Cell	K_D_ (µM)	N	ΔH (kJ/mol)	ΤΔS (kJ/mol)	ΔG (kJ/mol)
AKT_PH/TCL1A	7.5±1.0	0.7±0.3^a^	−60.9±2.5	−29.9	−31.0
AKT_PH/TCL1A:IκB	5.1±0.5	0.7±0.3^a^	−39.0±1.2	−9.4	−29.7
TCL1A/IκB	0.71±0.07	0.9±0.1	−53.2±0.5	−17.9	−35.3
TCL1A/IκB:RELA	≫50	n.d.	n.d.	n.d.	n.d.

n.d.: not determined.

a A 1∶1 stoichiometry can be deduced from our previous analyses [5].

**Figure 4:**
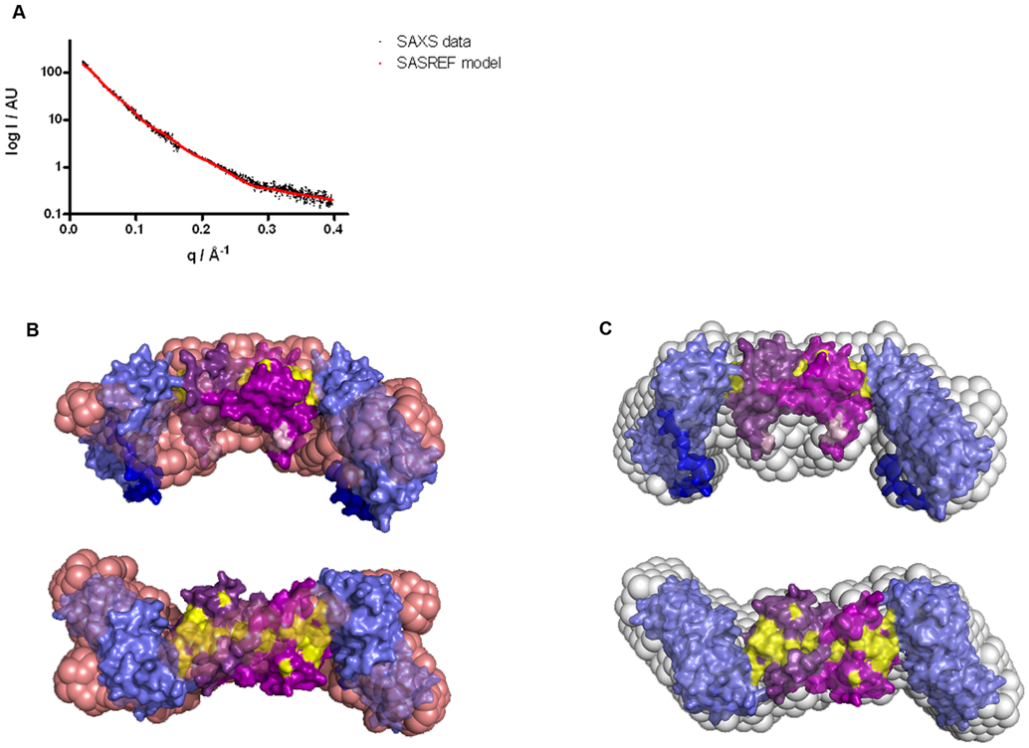
SAXS analysis. (a) SAXS curve (red) calculated from the molecular model depicted in b, fitted to SAXS data (black). (b) Best-scored SASREF model (surface representation). The TCL1A dimer (taken from PDB entry 1JSG [14]) is shown in two shades of violet. The AKT_PH binding sites of TCL1A (determined previously by NMR mapping [5]) are coloured yellow. IκB_ank (taken from PDB entry 1NFI [12]) is shown in blue. The disordered 20 C-terminal tail residues of IκB_ank (dark blue), and 7 N-terminal residues of TCL1A (light pink) were not included in the crystal structures. These fragments were modelled placed by SASREF as described in the Materials and Methods section. Because the dimensions of the *ab initio* GASBOR envelope (pink spheres) suggested that the 20 residues C-terminal tail residues of IκB_ank are pinned back onto the ankyrin repeats, this constraint was used in SASREF runs. We speculate that hydrophobic tail residues (such as F295 and F298) may cover hydrophobic surfaces on the ankyrin repeats that are also used by NF-κB. (c) The SASREF model is superimposed onto the ‘filtered’ average envelope (grey spheres) which was calculated by DAMAVER (file ‘damfilt’) from the 20% top SASREF solutions. The superimposition in DAMAVER was achieved allowing use of enantiomers. At the low resolution used, models where the C-terminus of the left IκB_ank molecule points down, and the right one up (using the orientation of the lower panel) resemble enantiomers of the model displayed. Since SAXS is unable to distinguish between enantiomers, models with both IκB_ank orientations were found among the top 20% best scored SASREF models; the orientation of the model displayed is used by 70% of the top 20% solutions, and may therefore be more likely.

In the absence of successful TCL1A∶IκB crystallisation trials, we used small angle X-ray scattering (SAXS) to obtain structural information on the complex. Guinier analysis and data processing by GNOM [13] established a radius of gyration of 4.5±0.2 nm and a maximum diameter of 15.0±0.5 nm. The P(r) distance distribution obtained for TCL1A∶IκB_ank^7CS^ displayed a triangular shape indicative for an elongated particle (Figure S3a). Initial *ab initio* shape calculations with DAMMIN [13] without symmetry constraints yielded elongated particles with a volume of 12.7×10^4^ Å^3^, comparable to the one of a 2×(TCL1A∶IκB_ank) complex (11.0×10^4^ Å^3^, calculated without hydration shell). The Z-shape of these reconstructions was suggestive of a P2 symmetry (data not shown). Because TCL1A forms stable dimers in solution [5], [14], we subsequently used P2 symmetry for iterative *ab initio* shape reconstructions with the program GASBOR [15]. The best scored *ab initio* structure out of 40 individual GASBOR runs fitted data between q = 0.02 and 0.45 Å^−1^ with a χ value of 1.02 (CRYSOL [16]; Figure S3b). This SAXS envelope is composed of a trapeze-shaped central domain, and two laterally attached rod-like domains (Figure 4b). The dimensions of the central and rod-like domains correspond to those of the crystal structures of the TCL1A dimer [14] and IκB_ank monomer [11], [12], respectively. The *ab inito* shape suggested that TCL1A binds either to the N- or C-terminal ankyrin repeats of IκB_ank. Since the C-terminal sixth ankyrin repeat of IκBα was dispensable for binding (Figure 1d), our analysis supports that TCL1A principally contacts the first N-terminal ankyrin repeats of IκBα.

We next used the crystal structures of TCL1A and IκB_ank to perform SAXS-guided rigid body docking with the program SASREF [13]. The information that TCL1A binds to the N- and not the C-terminal of IκB_ank was used as a constraint. The best-scored 20 structures out of 100 individual Monte-Carlo-based simulated annealing calculations were averaged using DAMAVER [17]. The representative structure determined by DAMAVER, which was also the one with the best fit of all SASREF runs (χ fit to data between q = 0.02 and 0.40 Å^−1^ was 1.02; Figure 4a), corresponded well to the *ab initio* GASBOR envelope (Figure 4b). Rg and Dm values for this structure (42.3 Å and 144.2 Å, respectively, calculated without hydration shell) were in good agreement with values obtained from GNOM and Guinier analysis for the hydrated particle (4.5±0.2 nm and 15.0±0.5 nm).

Our structural analysis suggests that TCL1A uses a binding site roughly opposite to its dimerisation site to interact with the first one or two ankyrin repeats of IκB_ank. Thus TCL1A may compete with helices αA and αB of RELA [11], [12]. The IκB_ank binding site is close to, but not overlapping with, the AKT_PH binding surface on TCL1A [5], corroborating that TCL1A can bind IκB and AKT simultaneously.

## Discussion

Several lines of evidence suggest an AKT-independent action of TCL1A on NF-κB pathways [9], [10], [18]. We here sharpened this picture by showing that TCL1A and the NF-κB inhibitor IκB associate *in vitro*, in yeast-two-hybrid systems, and when transiently overexpressed in 293 cells. We also showed *in vitro* that TCL1A competed with NF-κB for binding to IκB, suggesting that TCL1A interferes with the inhibitory interaction between IκB and NF-κB. Since TCL1A binds to the same first two ankyrin repeats of IκB which also interact with helices αA and αB of RELA [11], [12], it is likely that TLC1A and RELA use overlapping binding sites on IκB. However, we can not exclude that IκB binding to one partner causes conformational changes (such as a slight change in the curvature of the ankyrin repeats) that affect allosterically the (distant) binding site of the other partner.

On a cellular level, the effect expected to arise from the competition of TCL1A and NF-κB for IκB would be an AKT-independent NF-κB-activation by TCL1A. This was indeed recently observed by Pekarsky et al. [10]. These authors reported that this effect involves the association between TCL1A and p300. The molecular details of the association between TCL1A and p300 remain elusive. Since both proteins are multivalent adaptor proteins, sequential or concomitant interactions of TCL1A with IκB are not excluded, and may be necessary for activation of NF-κB.

The description of several AKT-independent TCL1A targets suggests that TCL1A affects a number of alternative and interconnected signalling pathways ([10], [18], and this report). Indeed, depending on the cell type and experimental conditions, both NF-κB activation and inhibition by TCL1A were reported, as well as alternative, NF-κB independent routes (inhibition of PKCθ and ERK activation, or of AP-1 transcriptional activity) [10], [18]. Thus, TCL1A increasingly appears as a polyvalent adaptor protein, whose cellular action is dramatically affected by its sub-cellular concentration and the availability of potential targets.

The affinity we measured between IκB and TCL1A was about 1,000 fold weaker than the one previously reported between IκB and NF-κB [19]. However, since only a minor fraction of cytoplasmic NF-κB is enough to trigger transcriptional activation [20], even weak competition by TCL1A is expected to alter gene expression through NF-κB. Yet this effect will be caused by only a negligible fraction of cellular IκB and NF-κB, which may explain why we were unable to detect significant amounts of TCL1A-IκB complexes in absence of transient overexpression, as searched for in 697 and Sup-T11 TCL1-positive leukaemic cell lines (data not shown). In addition, a biologically significant effect on the IκB∶NF-κB complex is likely to require TCL1A over-expression following chromosomal translocation, and/or additional factors, such as AKT, p300, or factors associated with a particular cell activation level. Importantly, TCL1A also needs co-stimulatory signals to induce AKT activation (possibly the association of AKT_PH with PIP_3_ [2], [3]), and the requirement of co-activators may constitute an additional control mechanism for TCL1A.

Given that TCL1A binds IκB and AKT_PH simultaneously *in vitro*, TCL1A may act on IκB and AKT synergistically or independently. In its physiological location, the 5′ promoter region of the *TCL1A* gene contains, among others, an NF-κB-responsive *cis*-regulatory element [7]. Through the action of TCL1A on IκB or AKT, this element may produce a positive feed-back loop to enhance TCL1A expression. Thus, the association between TCL1A and IκB could contribute to transcriptional regulation of TCL1A.

Future research will be necessary to clarify the *in vivo* significance and role of the TCL1A-IκB interaction for oncogenesis. However, given that TCL1A appears to be a polyvalent adaptor protein with multiple cellular partners, it will be far from trivial to design a cell-based assay that allows observing a molecular effect based solely on this TCL1A-IκB interaction. Moreover, because only an extremely low fraction of cellular IκB∶NF-κB complexes is predicted to be affected by TCL1A, it will be difficult to use intracellular detection methods based on endogenous protein (for example co-localisation studies by immunofluorescence, or co-immunoprecipation experiments). The identification of potential binding surfaces by our structural characterisation of the TCL1A-IκB interaction may however help resolving these issues.

## Materials and Methods

### Yeast two-hybrid screening of TCL1A

The yeast two hybrid screening was performed as previously described [21]. Briefly the yeast reporter strain HF7c was transformed by the lithium acetate method with the bait plasmid pGBT10-TCL1A. After selection and transformation with a peripheral blood mononuclear cell cDNA library cloned into the pGAD plasmid, approximately 2.10^6^ clones were screened for HIS+βGAL+ colonies. Out of the 30 HIS+βGAL+ colonies sequenced, 28 corresponded to the 735 nucleotides at the 3′ end of IκBα transcripts.

### Binding analysis through retention by glutathione-sepharose-bound GST-TCL1A

Recombinant GST-TCL1A and GST-MTCP1 were expressed in *E. coli*, bound to glutathione-sepharose beads (Pharmacia), and placed into Handee Centrifuge Columns. Unbound material was removed by washing the beads four times with 100 column volumes of TN300 (10 mM Tris, pH 7.4 and 300 mM NaCl). Beads were incubated for 1 h at 4°C with 3 mg of AKT_PH, IκB_ank^7CS^, or the Drosophila Notch ankyrin-repeat protein and washed with 15 column volumes with TN300. The bound proteins were visualized using SDS PAGE gel under denaturing conditions. Alternatively, GST proteins bound to the beads were incubated with rabbit reticulocyte lysates containing [^35^S]-labeled translated IκBα or IκBα_1–253_ (T7 Quick Coupled Transcription/Translation System, Promega Madison, WI). Equal loading of the GST fusion proteins was confirmed by Coomassie staining. Input corresponds to 5% of the total protein content used for the pull-down. Bound IκBα and IκBα_1–253_ were recovered in SDS loading buffer, resolved by 12% SDS-PAGE and analyzed by autoradiography of dried gels.

### Co-immunoprecipitation assay

293 cells were transiently transfected with pCMV2-FLAG-TCL1A (Sigma-Aldrich, Saint Quentin Fallavier, France) and pCMV-IκBα, using the calcium phosphate precipitation technique as described [22]. Cell extracts were incubated with a mouse anti-Flag antibody (Sigma-Aldrich) for 16 h and then with protein G-Sepharose (Amersham Pharmacia Biotech) for an additional hour. The immunocomplexes were recovered by centrifugation, washed and resolved by SDS-PAGE. The immunoprecipitated proteins were revealed by immunoblotting and chemiluminescence.

### Western Blot analysis

For Western Blot analysis, the native gels were applied to a PolyScreen PVDF transfer membrane (NEN Life Science Products). Membranes were treated as suggested by the manufacturer, and incubated with mouse anti-IκBα antibody (1 h at room temperature), followed by membrane washing in the Tris-buffered saline. Membranes were then incubated with anti-mouse IgG linked to horseradish (30 min at room temperature). Membranes were washed following the manufacturer's directions, and ECL detection reagents were added. Kodak biomax light film was exposed to membranes for ∼25 s. The positions of the complexes formed are indicated.

### Recombinant protein production

The pET11a expression plasmid for human IκB_67–302_ (IκB_ank) was provided by G. Ghosh. The NF-κB family member RELA/p65, cloned into pGEX2T, was provided by M. Benkirane. IκB and RELA were expressed and purified as described [23], [24]. Human TCL1A and human AKTβ PH_1–118_ (AKT_PH) were produced as published [14], [25]. The Cys->Ser mutant IκB_67–302_^7CS^ (IκB_ank^7CS^) was obtained using Stratagene's QuikChange multi-site directed mutagenesis Kit. The purified recombinant ankyrin domain of the *Drosophila* signalling protein Notch was provided by J.-B. Rouget.

### PAGE gel shift assays

Undenatured (native) polyacrylamide gel electrophoresis (PAGE) was performed with purified recombinant proteins. Running gels contained 15% (w/v) polyacrylamide and 0.125 M Tris (pH 6.8). Stacking gels contained 5% (w/v) polyacrylamide and 0.375 M Tris (pH 8.8). For additional Western blot analysis, the native gels were applied to a PolyScreen PVDF transfer membrane (NEN Life Science Products). Membranes were treated as suggested by the manufacturer, and incubated with mouse anti-IκBα antibody (1 h at room temperature), followed by membrane washing in the Tris-buffered saline. Membranes were then incubated with anti-mouse IgG linked to horseradish (30 min at room temperature). Membranes were washed following the manufacturer's directions, and ECL detection reagents were added. Kodak biomax light film was exposed to membranes for ∼25 s.

### Isothermal titration calorimetry (ITC)

Purified TCL1A, IκB_ank^7CS^, RELA and AKT_PH were extensively dialysed in degassed ITC buffer. The TCL1A - IκB_ank^7CS^ titrations were carried out in a buffer similar to the SAXS measurement (10 mM Tris pH 7.4, 300 mM NaCl, 1 mM β-mercapto ethanol). All other titrations were carried out in 20 mM sodium phosphate pH 7.1, 300 mM NaCl, 2 mM EGTA and 1 mM β-mercapto ethanol. ITC was performed at 20°C with a VP-ITC microcalorimeter from MicroCal Incorporated. For the AKT_PH –TCL1A titration, TCL1A was placed in the 1.4 mL sample cell at a concentration of 40 µM, and AKT_PH (490 µM) was injected using 10–15 µl injections every 300 s. For the IkBα–TCL1A titration, IκB_ank^7CS^ (45 µM) was kept in the sample cell, and TCL1A (470 µM) was injected. Competition experiments were performed by placing equimolar amounts of TCL1A and IκB_ank^7CS^ in the sample cell, and injecting AKT_PH. For all protein-protein titrations, control experiments were performed where the corresponding protein was injected in buffer alone. The resulting heats were subtracted from the protein-protein titrations prior to analysis. Data were fitted with Microcal Origin software.

### SAXS analysis

Data used for this SAXS analysis were collected at beamline X33, at DESY, EMBL, Hamburg, at 10°C, λ = 1.5 Å. Additional preliminary data were collected at the ESRF beamline ID14-3. For SAXS analysis of the TCL1A∶IκB_ank^7CS^ complex, the partners were pre-purified individually, then mixed in a 1∶1 ratio, and applied to a S200 size-exclusion column (Pharmacia). Only eluted fractions corresponding to the 2×2 TCL1A∶IκB_ank^7CS^ dimer were used for SAXS. Samples were kept in 10 mM Tris, pH 7.4, 300 mM NaCl, 1 mM β-mercapto ethanol and supplemented with 5 mM DTT prior to exposure. Data collected on purified TCL1A∶IκB_ank^7CS^ at 1 and 7 mg/ml were merged to combine the aggregate-free low resolution-part of the 1 mg/ml scattering curve with the reduced noise at high resolution of the 7 mg/ml data. Data analysis, *ab initio* shape calculations, and rigid body docking were performed using programs by S. Svergun and colleagues (PRIMUS, GNOM, DAMMIN, CRYSOL, DAMAVER and SASREF [13], [16], [26]–[28]). For GASBOR different q ranges (0.020–0.45 Å^−1^ or 0.028–0.40 Å^−1^) gave very similar results. For SASREF rigid body docking, P2 symmetry was used. The TCL1A dimer was obtained from the PDB entry 1JSG [14]), and this dimeric arrangement was kept fixed during rigid body docking. IκB_ank was obtained from PDB entry 1NFI [12]. The regions not included into the crystallographic models (7 N-terminal residues for TCL1A, and 20 C-terminal residues for IκB_ank) were modelled as extended unstructured regions and included as rigid fragments in SASREF modelling. For this, the 20 C-terminal residues of IκB_ank were divided into two rigid 10-residue fragments. The fragments forming one polypeptide chain were connected using the constraint that connecting residues lie within 4.0 Å of each other. SASREF models for different q ranges (0.020–0.40 Å^−1^ or 0.028–0.40 Å^−1^) were very similar.

## Supporting Information

Figure S1 Binding assays. (a) Glutathione-sepharose beads containing GST_TCL1A alone, or GST_TCL1A and indicated ligands, were washed and analysed by SDS PAGE gels as described in Supplementary Methods. Notch ank has the same molecular weight (26 kDa) as IκB_ank. (b) Native gel shift assays, stained with coomassie blue, or (c) revealed by Western blotting, using a mouse anti-IκBα antibody.
(0.30 MB TIF)

Figure S2 Supporting ITC data. All experiments were performed at 20°C. Upper panels show the binding isotherms (black) and their control experiments (blue) where the syringe content was injected into the cell containing only buffer. Lower panels show integrated heats, after subtraction of heats from control experiments (dots). The black lines represent least-square fits of data. (a) ITC binding isotherm obtained when AKT_PH was injected into the TCL1A-containing cell. (b) ITC binding isotherm for AKT_PH injected into the cell containing the TCL1A:IκB_ank7CS complex.
(0.19 MB DOC)

Figure S3 SAXS Distance distribution [P(r)] and SASREF results. (a) P(r) distance distribution, and (b) fit of best GASBOR model to data (q = 0.02–0.45 Å^−1^).
(0.06 MB DOC)
