# Supplementary figures and images for: The TCL1A Oncoprotein Interacts Directly with the NF-κB Inhibitor IκB
Source: PLoS One. 2009 Aug 10;4(8):e6567. doi: 10.1371/journal.pone.0006567 (PMC2718698; doi:10.1371/journal.pone.0006567)

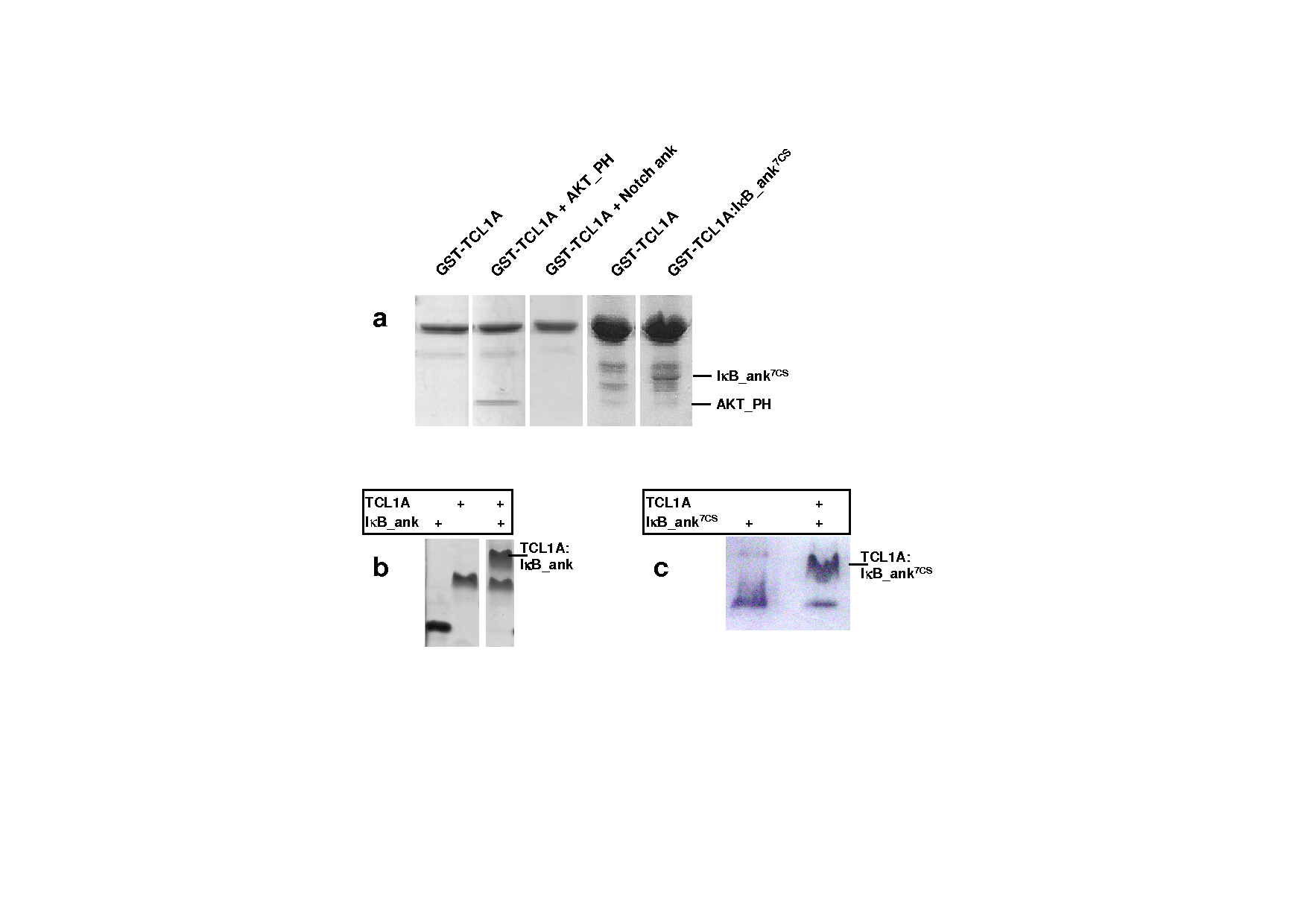

Supplement: Figure S1 — Binding assays. (a) Glutathione-sepharose beads containing GST_TCL1A alone, or GST_TCL1A and indicated ligands, were washed and analysed by SDS PAGE gels as described in Supplementary Methods. Notch ank has the same molecular weight (26 kDa) as IκB_ank. (b) Native gel shift assays, stained with coomassie blue, or (c) revealed by Western blotting, using a mouse anti-IκBα antibody. (0.30 MB TIF) [file pone.0006567.s001.tif]

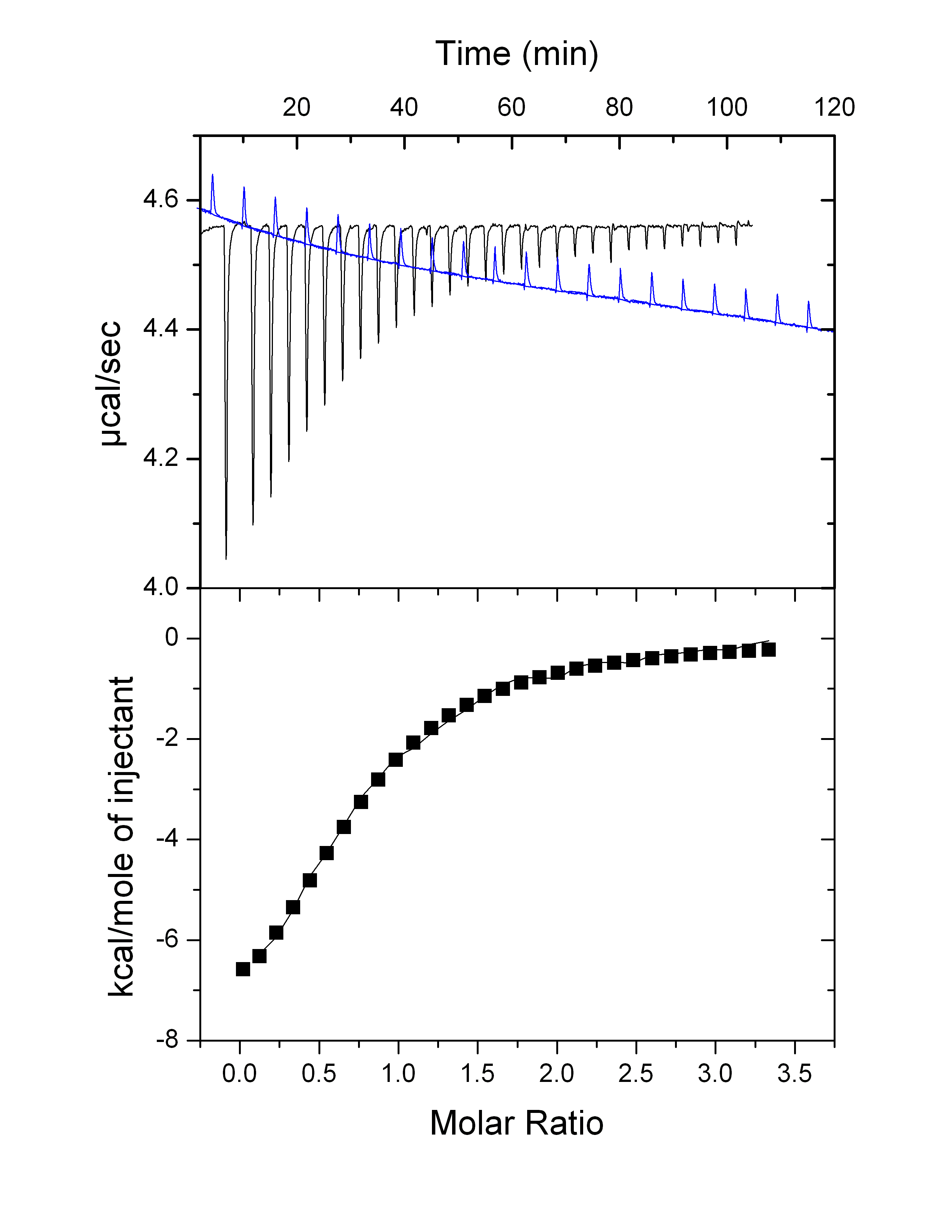

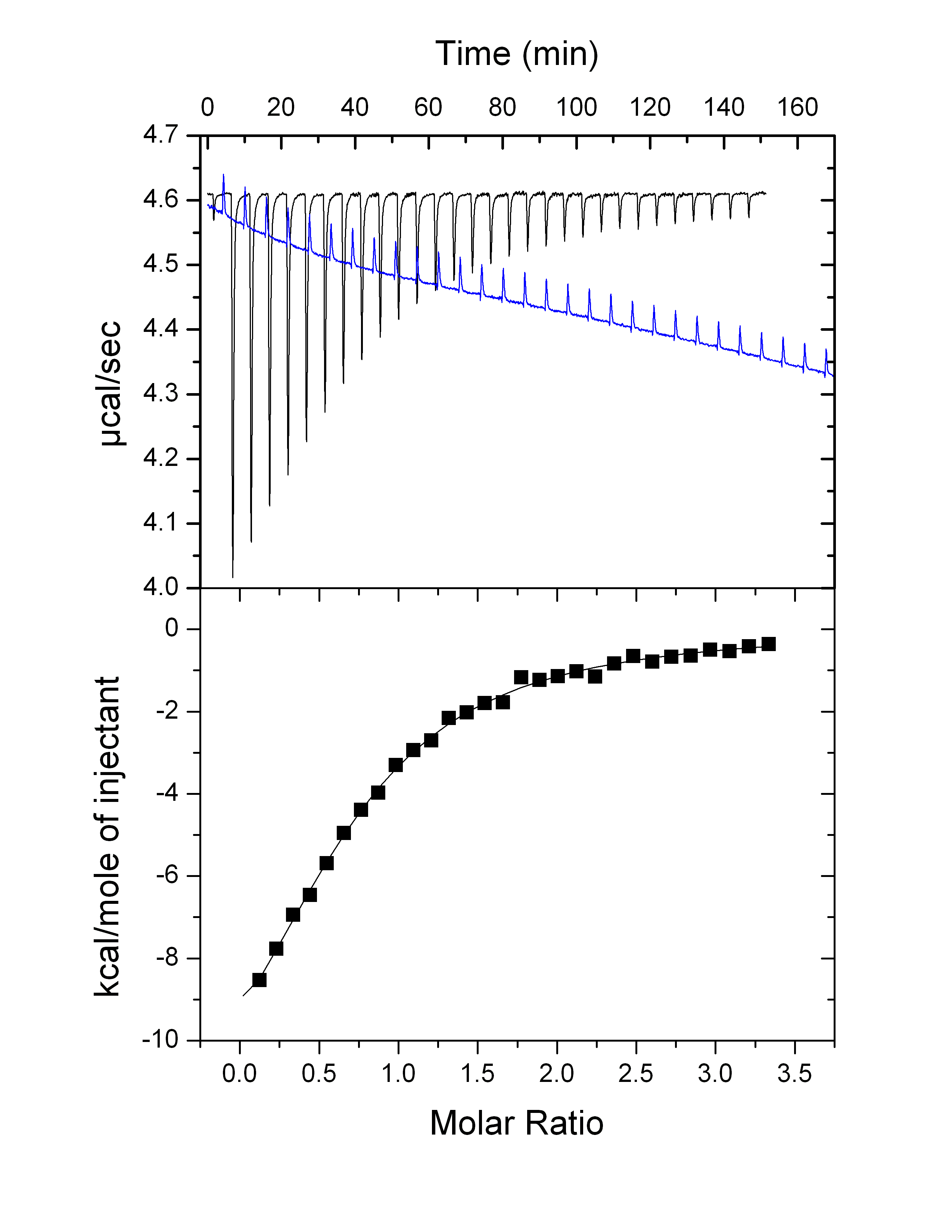


**a**

**b**

Supplement: Figure S2 — Supporting ITC data. All experiments were performed at 20°C. Upper panels show the binding isotherms (black) and their control experiments (blue) where the syringe content was injected into the cell containing only buffer. Lower panels show integrated heats, after subtraction of heats from control experiments (dots). The black lines represent least-square fits of data. (a) ITC binding isotherm obtained when AKT_PH was injected into the TCL1A-containing cell. (b) ITC binding isotherm for AKT_PH injected into the cell containing the TCL1A:IκB_ank7CS complex. (0.19 MB DOC) [file pone.0006567.s002.doc]

**
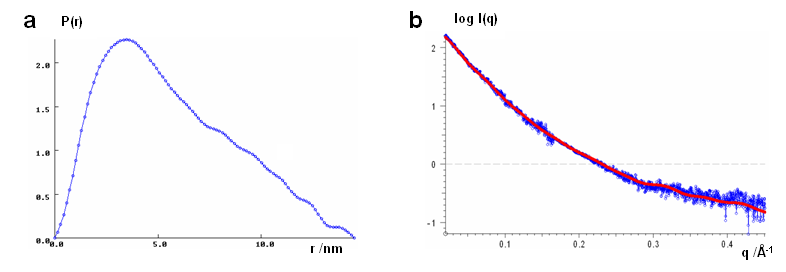
**

Supplement: Figure S3 — SAXS Distance distribution [P(r)] and SASREF results. (a) P(r) distance distribution, and (b) fit of best GASBOR model to data (q = 0.02–0.45 Å−1). (0.06 MB DOC) [file pone.0006567.s003.doc]
